# Supplementary material for: Genomic alterations and transcriptional phenotypes in circulating free DNA and matched metastatic tumor
Source: Genome Med. 2025 Feb 25;17:15. doi: 10.1186/s13073-025-01438-4 (PMC11863907; doi:10.1186/s13073-025-01438-4)
Supplement: Supplementary file 1 — Additional file 1: Fig. S1. cfDNA and tumor sampling schema. Fig. S2. Copy number-predicted tumor purity correlated with cfDNA tumor fraction. Fig. S3. Mutation profiles of small cell lung cancer (SCLC) circulating free DNA (cfDNA). Fig. S4. Circulating cell free DNA (cfDNA) fragment length analysis. Fig. S5. Circulating cell free DNA (cfDNA) tracks the clinical course. Fig. S6. Dynamic changes of circulating cell-free DNA (cfDNA) occupancy around transcription factor binding sites. Fig. S7. A Kaplan-Meier curve of overall survival (OS) in patients with high vs. low predicted REST expression. Fig. S8. Correlation of cfDNA tumor fraction and nucleosome occupancy at binding sites of a transcription factor ASCL1. Table S1: Comparisons of clinical characteristics between patients with high vs. low pre-treatment circulating cell-free (cfDNA) tumor fraction. Table S2: Multivariate Cox regression analysis of progression-free survival (PFS) between patients with high vs. low pre-treatment circulating cell-free DNA (cfDNA) tumor fraction. Table S3: Multivariate Cox regression analysis of overall survival (OS) between patients with high vs. low pre-treatment circulating free DNA fraction. Table S4: Names of transcription factors (TFs) and the number of binding sites for TFs enriched in genomic regions with differential circulating cell-free DNA (cfDNA) occupancy post-treatment vs. pre-treatment. [file 13073_2025_1438_MOESM1_ESM.docx]

**Genomic alterations and transcriptional phenotypes in circulating free DNA and matched metastatic tumor**

**Supplementary figures and tables**

Nobuyuki Takahashi^1,2, 3^*, Lorinc Pongor^1^*, Shivam P. Agrawal^4^, Mariya Shtumpf^4^, Ankita Gurjar^4^, Vinodh N. Rajapakse^1^, Ahmad Shafiei^5^, Christopher W. Schultz^1^, Sehyun Kim^1,6^, Diana Roame^7^, Paula Carter^7^, Rasa Vilimas^1^, Samantha Nichols^1^, Parth Desai^1^, William Douglas Figg^7^, Mohammad Bagheri^5^, Vladimir B. Teif^4^**, Anish Thomas^1^**

^1^Developmental Therapeutics Branch, Center for Cancer Research, National Cancer Institute, Bethesda, USA

^2^Medical Oncology Branch, Center Hospital, National Center for Global Health and Medicine, Tokyo, Japan

^3^Department of Medical Oncology, National Cancer Center East Hospital, Kashiwa, Japan

^4^School of Life Sciences, University of Essex, Colchester, UK

^5^Department of Radiology and Imaging Sciences, Center for Cancer Research, National Cancer Institute, Bethesda, USA

^6^Department of Internal Medicine, Seoul National University Bundang Hospital, Seoul National University College of Medicine, Seongnam, Korea

^7^Genitourinary Malignancies Branch, Center for Cancer Research, National Cancer Institute, Bethesda, USA

*Equal contributions

**Corresponding authors

Lead contact: Anish Thomas, Center for Cancer Research, National Cancer Institute, Building 10 Room 4-5330, Bethesda, MD 20892; Ph: 240-760-7343; Fax: 954-827-0184; Email: anish.thomas@nih.gov

**
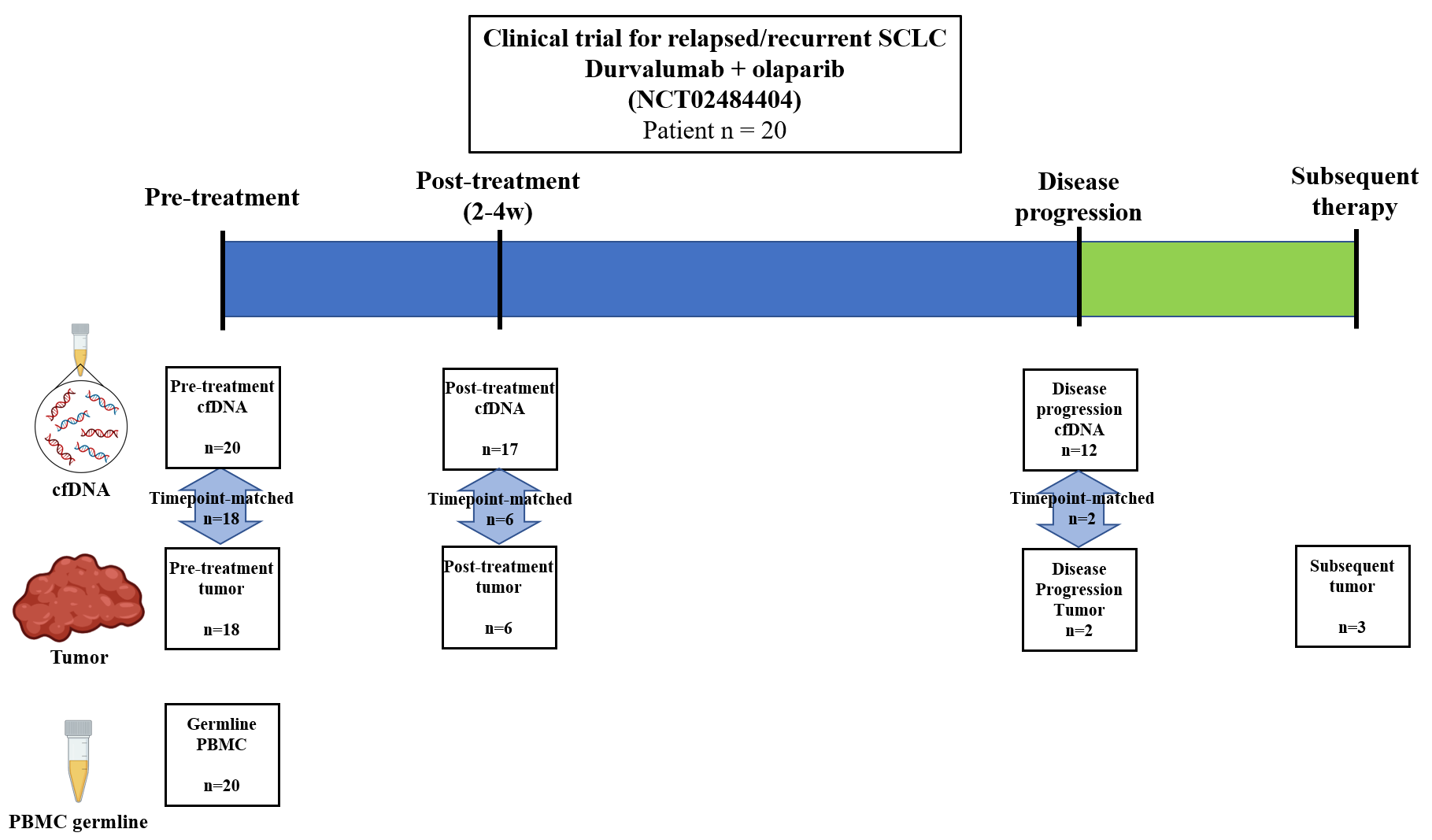
**

**Fig. S1. cfDNA and tumor sampling schema**

Abbreviations: SCLC: small cell lung cancer; w: week; cfDNA: circulating free DNA; PBMC: peripheral blood mononuclear cell.

**
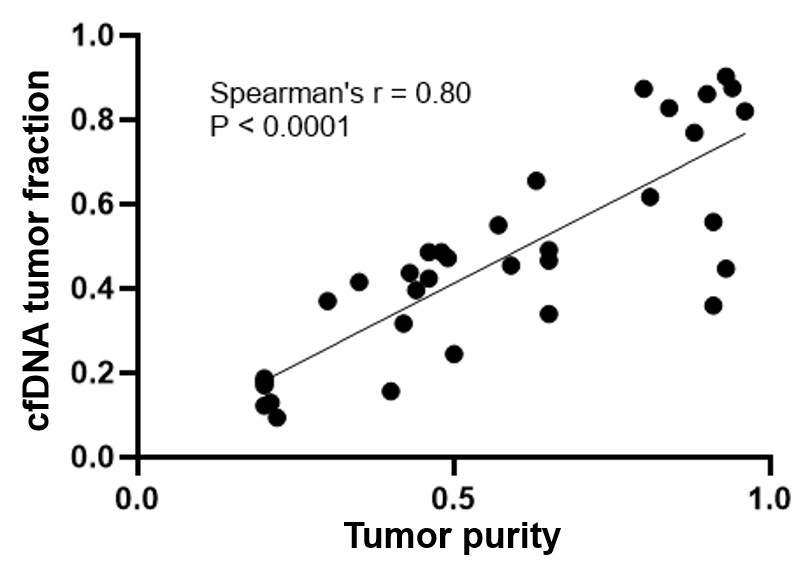
**

**Fig. S2. Copy number-predicted tumor purity correlated with cfDNA tumor fraction**

A: A correlation between tumor fraction estimated by *ichorCNA* (1) and tumor purity estimated by *sclust* (2) and *sequenza* (3)

**
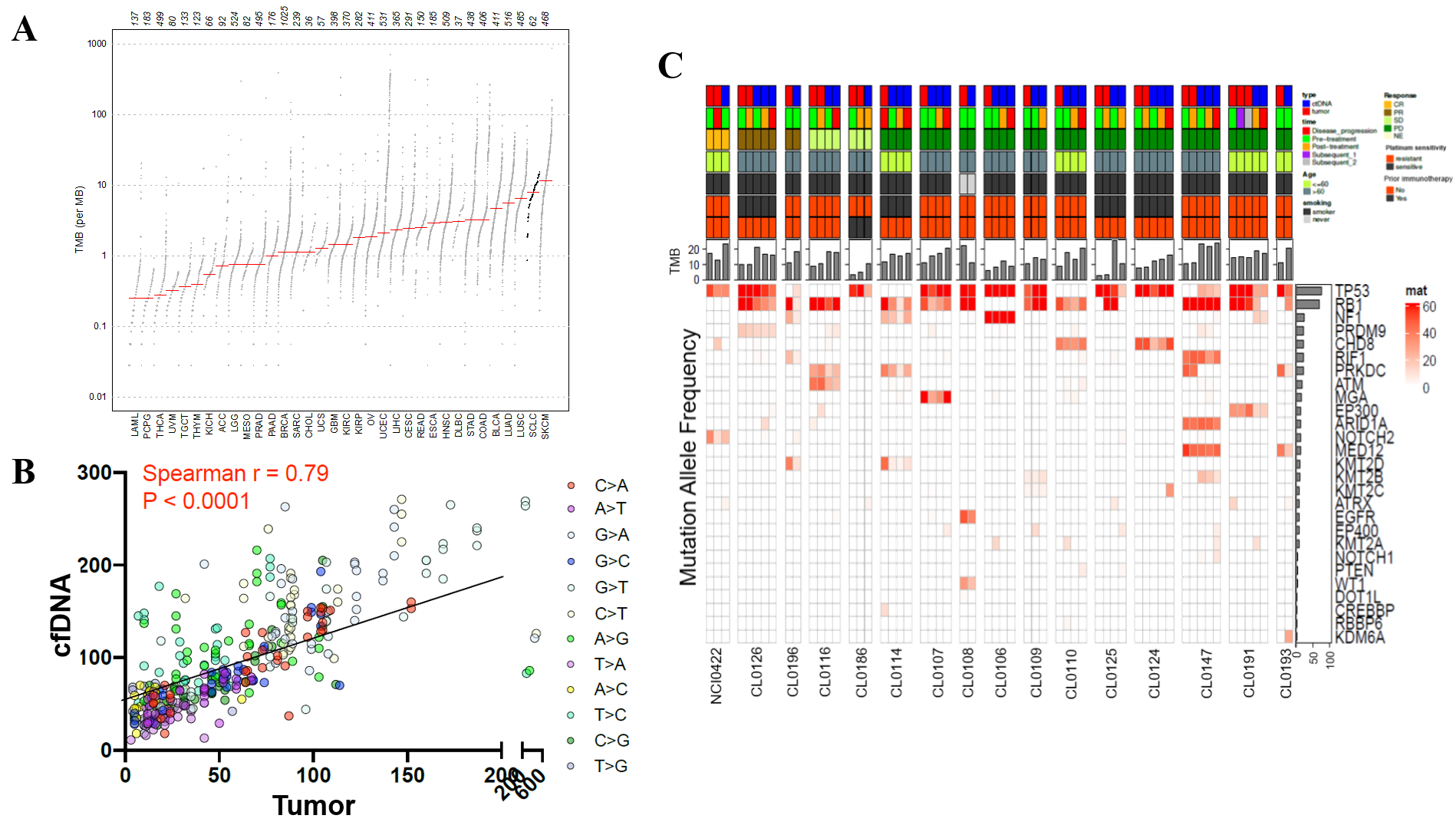
**

**Fig. S3. Mutation profiles of small cell lung cancer (SCLC) circulating free DNA (cfDNA)**

A: TMB comparison of SCLC samples compared to the TCGA cohort

B: A correlation of the numbers of SNVs between cfDNA and tumor samples

Abbreviations for cancer types in TCGA are available in https://gdc.cancer.gov/resources-tcga-users/tcga-code-tables/tcga-study-abbreviations.

C: Clinical characteristics, TMB, and MAFs of SNVs in cfDNA and tumor

Abbreviations: SNV: somatic nucleotide variant; TMB: tumor mutational burden; MAFs: mutation allele frequency; TCGA: the Cancer Genome Atlas; cfDNA: circulating free DNA.


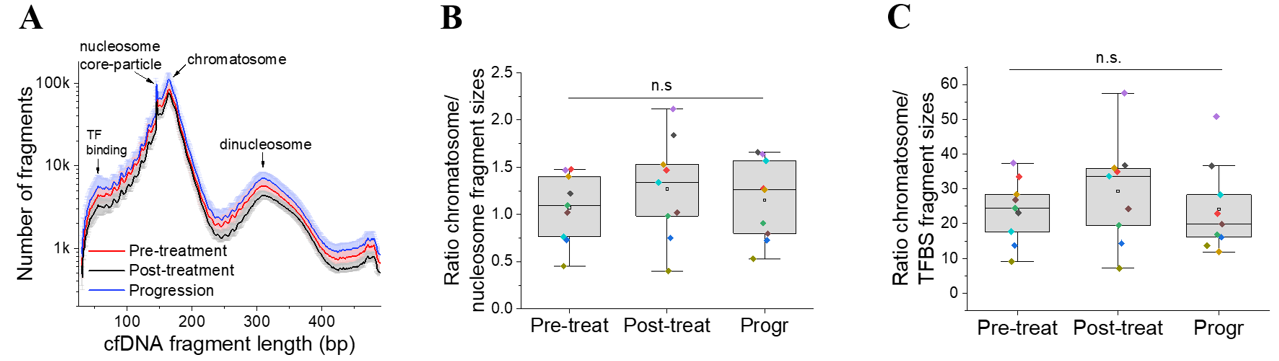


**Fig. S4. Circulating cell free DNA (cfDNA) fragment length analysis**

A: The average distributions of cfDNA fragment sizes showing the peaks corresponding to DNA protection by the chromatosome (~165 bp), nucleosome core particle (~150 bp) and TF binding (~50 bp)

B: Dynamics of the ratio of the numbers of DNA fragments with sizes characteristic for protection by chromatosome (~165 bp) versus nucleosome core-particle (~150 bp) over treatment time course

C: Dynamics of the ratio of the numbers of DNA fragments with sizes characteristic for protection by chromatosome (~165 bp) versus TF-binding (~50 bp) over treatment time course

n.s.: not significant, P value > 0.05 by Wilcoxon signed rank test of each pair comparison followed by Benjamini and Hochberg corrections.

Abbreviations: cfDNA: circulating cell free DNA; bp: based pairs; Pre-treat: pre-treatment; Post-treat: post-treatment; Progr: disease progression.


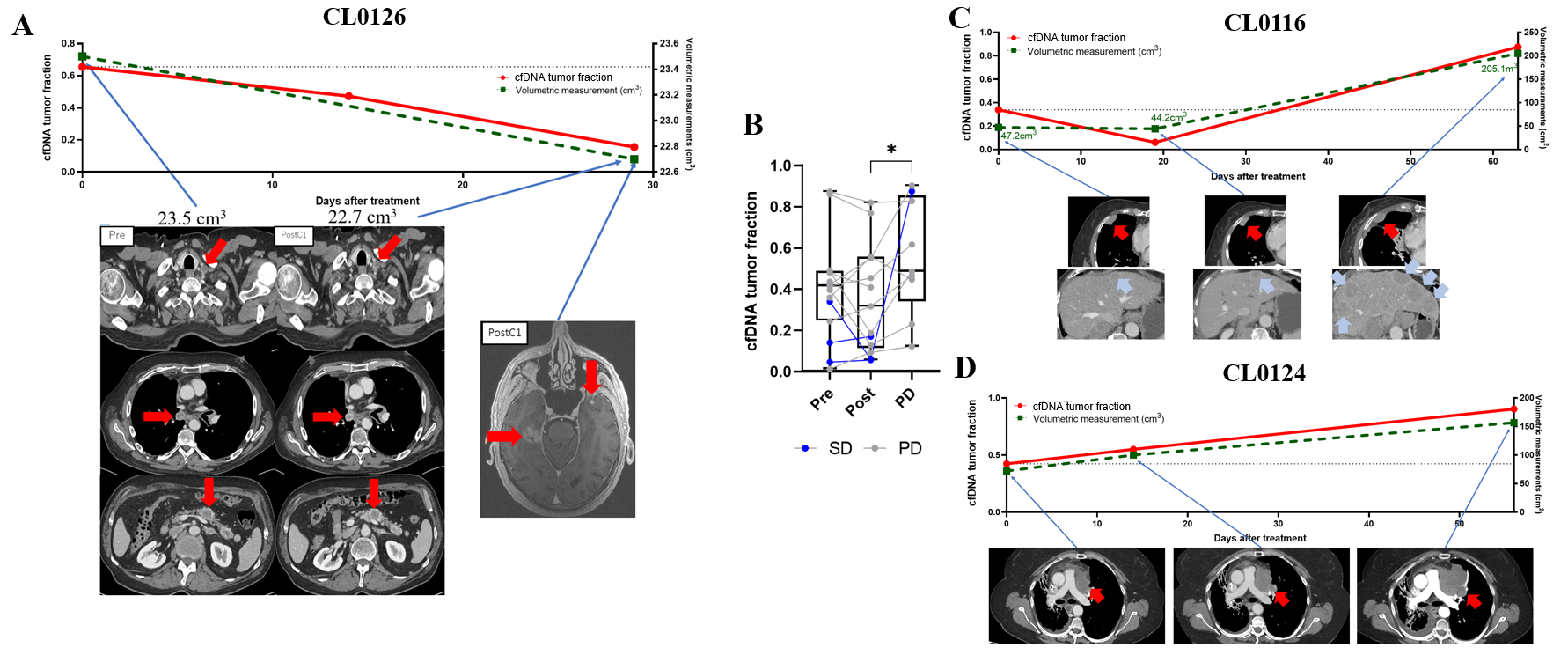


**Fig. S5. Circulating cell free DNA (cfDNA) tracks the clinical course**

A: Changes of cfDNA tumor fraction (red solid line, left y-axis) and radiological volumetric tumor measurement (green dash line, right y-axis) through treatment time course in a patient who had minor reduction in size of thoracic and abdominal lesions followed by brain only progression (CL0126)

Red arrows in CT images indicate left supraclavicular lymph node metastasis (left top), right mediastinal lymph node metastasis (left middle), pancreatic metastasis (left bottom), and brain metastases (right).

We modified best response of the CL0126 as “partial response”, given that the patient experienced a substantial decrease of total tumor burden even if the patient developed new brain metastasis, considering the ctDNA recapitulate genomic characteristics systemically.

B: Changes of cfDNA tumor fractions in patients who had SD (blue lines) or PD (gray lines) as best response

*: P < 0.05 by Wilcoxon signed rank test

C: Changes of cfDNA tumor fraction (red solid line, left y-axis) and radiological volumetric tumor measurement (green dash line, right y-axis) through treatment time course in a patient who had minor tumor shrinkage followed by disease progression (CL0116)

Red and light blue arrows in CT images indicate a pleural lesion and hepatic lesions, respectively.

D: Changes of cfDNA tumor fraction (red solid line, left y-axis) and radiological volumetric tumor measurement (green dash line, right y-axis) through treatment time course in a patient who had PD as best response (CL0124)

Red arrows in CT images indicate a mediastinal mass.

Abbreviations: SCLC: small cell lung cancer; SD; stable disease; PD: progressive disease; CT: computed tomography.


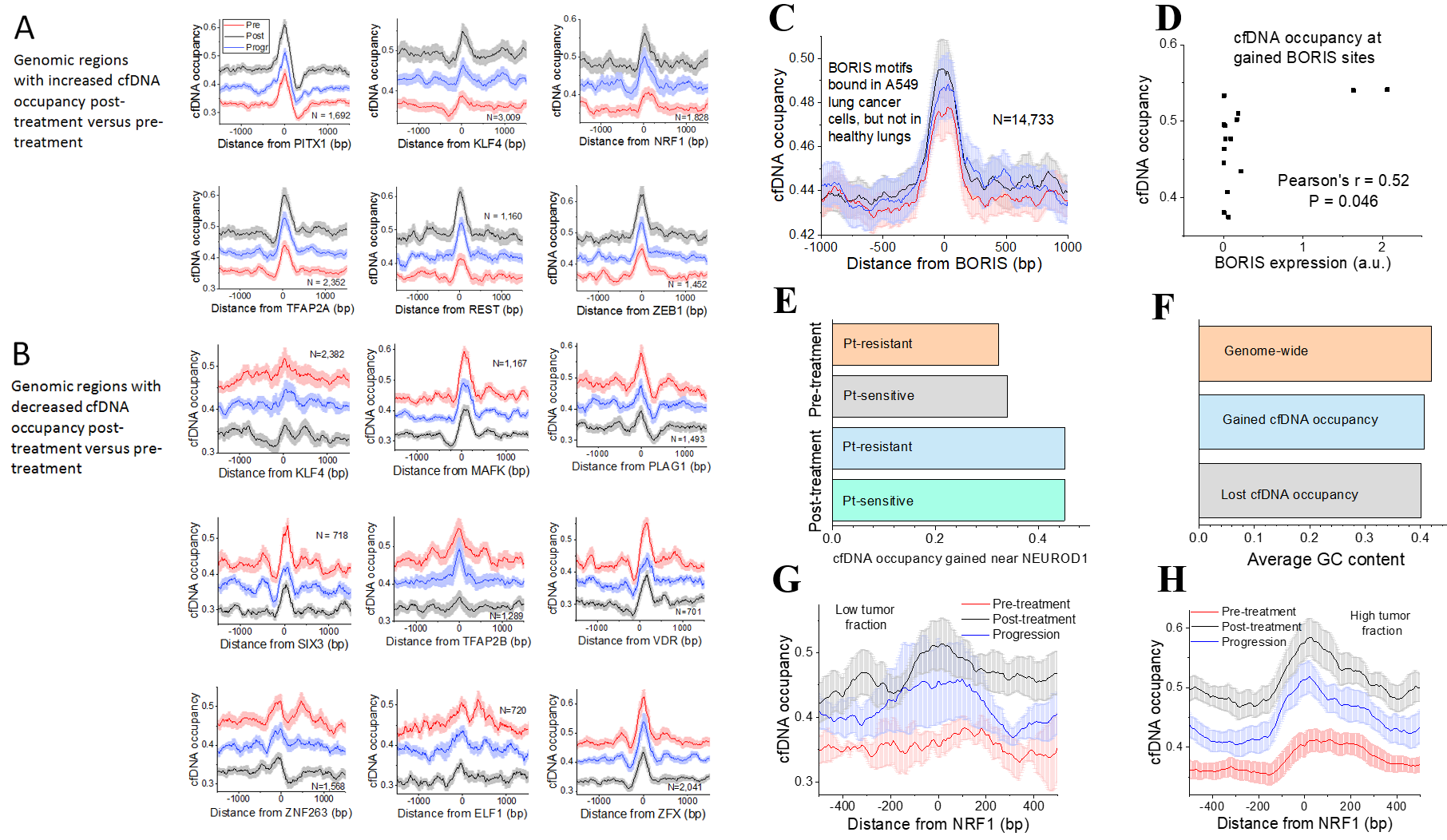


**Fig. S6. Dynamic changes of circulating cell-free DNA (cfDNA) occupancy around transcription factor binding sites**

A, B: Aggregate profiles in cfDNA around transcription factor binding sites in regions where cfDNA occupancy (A) increased and (B) decreased post-treatment vs pre-treatment

Red, black, and blue lines indicate pre-treatment, post-treatment, and at disease progression, respectively.

C: cfDNA occupancy profile around binding sites of *CTCFL* (*BORIS*) determined with ChIP-seq in A549 SCLC cell line, which are not bound by *CTCF* in healthy lung cells.

D: A correlation between cfDNA occupancy at *CTCFL* (*BORIS*) binding sites from panel C with *CTCFL* expression pre-treatment in corresponding tumors.

Binding of *CTCF* and *CTCFL* (*BORIS*) is defined based on ChIP-seq in SCLC A549 cell line and healthy donor cells retrieved from a previous report (4).

E: cfDNA occupancy at binding sites of *NEUROD1* in platinum-resistant and platinum-sensitive patients pre-treatment as well as platinum-sensitive and platinum-resistant patients post-treatment, inside genomic regions that gained cfDNA occupancy post-treatment vs pre-treatment.

F: Average GC content inside cfDNA fragments genome-wide and in regions which increased/decreased cfDNA occupancy post-treatment.

G, H: cfDNA occupancy at *NRF1* binding sites from Figure 6A calculated separately in samples with cfDNA tumor fraction <10% (G) and >10% (H).


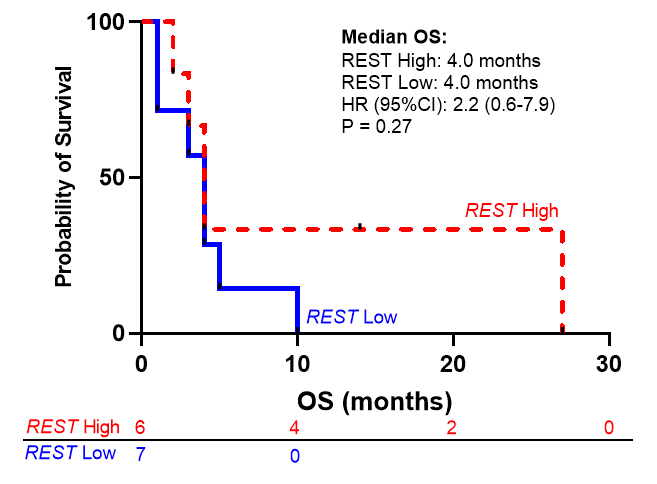


**Fig. S7. A Kaplan-Meier curve of overall survival (OS) in patients with high vs. low predicted REST expression**

High vs. low predicted REST expression is defined as higher or lower than median predicted REST expression among 13 patients whose pre-treatment cfDNA is successfully processed for the TFBS analysis. Higher predicted REST expression was defined as lower read depth and vice versa, given that higher read depth indicates less TF binding, predicting less gene expression. P value is evaluated by Log-rank test.

Abbreviations: HR: hazard ratio; CI: confidence interval; cfDNA: circulating free DNA; TFBS: transcription factor binding site.

**
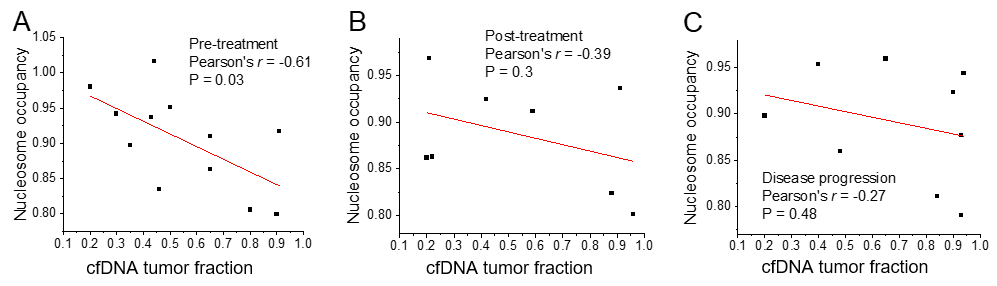
**

**Fig. S8. Correlation of cfDNA tumor fraction and nucleosome occupancy at binding sites of a transcription factor ASCL1**

A: Pre-treatment (as in Figure 6N in the main manuscript). Pearson’s r = -0.61, P = 0.03.

B: Post-treatment. Pearson’s r = -0.39, P = 0.30

C: Disease progression. Pearson’s r = -0.27, P = 0.48

**Table S1: Comparisons of clinical characteristics between patients with high vs. low pre-treatment circulating cell-free (cfDNA) tumor fraction**

|  | All patients (n=20) | cfDNA Tumor fraction low (n=10) | cfDNA Tumor fraction high (n=10) | P value |
| --- | --- | --- | --- | --- |
| Age at inclusion  (years) | 63 (56-69) | 61 (54-69) | 63 (59-64) | 0.92 |
| Sex  (male/female) | 9/11  (45.0%/55.0%) | 3/7  (30.0%/70.0%) | 6/4  (60.0% vs. 40.0%) | 0.37 |
| Ethnicity  (White/Black/Asian) | 18/1/1  (90.0%/5.0%/5.0%) | 8/1/1  (80.0%/10.0%/10.0%) | 10/0/0  (100%/0%/0%) | 0.47 |
| VALG stage  at diagnosis  (Limited/Extensive) | 7/13  (35.0%/65.0%) | 4/6  (40.0%/60.0%) | 3/7  (30.0%/70.0%) | 1.00 |
| ECOG PS  (0/1/2) | 1/18/1  (5.0%/90.0%/5.0%) | 1/9/0  (10.0%/90.0%/0%) | 0/9/1  (0%/90.0%/10.0%) | 1.00 |
| Smoking  (Never/former or current) | 2/18  (10.0%/90.0%) | 1/9  (10.0%/90.0%) | 1/9  (10.0%/90.0%) | 1.00 |
| Platinum sensitivity*  (Sensitive vs. resistant) | 6/14  (30.0%/70.0%) | 2/8  (20.0%/80.0%) | 4/6  (40.0%/60.0%) | 0.63 |

Data are presented as n (%) or median (range). P values for continuous and categorical values are evaluated by Mann-Whitney U test and Fisher’s exact test, respectively.

*: Platinum–sensitive was defined as disease progression ≥ 90 days after first-line platinum–based chemotherapy, and platinum-resistant as disease progression < 90 days or during first-line chemotherapy.

Abbreviations: ECOG PS: Eastern Cooperative Oncology Group performance status; VALG: Veterans Administration Lung Study Group.

**Table S2: Multivariate Cox regression analysis of progression free survival (PFS) between patients with high vs. low pre-treatment circulating cell-free DNA (cfDNA) tumor fraction**

| **Factors** | **Hazard ratio** | **Standard error** | **P value** | **95% CI** |
| --- | --- | --- | --- | --- |
| Age at inclusion (years) | 0.99 | 0.03 | 0.70 | 0.92–1.06 |
| Sex  (ref: male = 1) | 0.55 | 0.29 | 0.27 | 0.19–1.57 |
| Platinum sensitivity  (ref: platinum sensitive = 1) | 0.93 | 0.52 | 0.90 | 0.32–2.77 |
| cfDNA tumor fraction  (ref: low = 1) | 9.29 | 9.44 | 0.030 | 1.27–68.10 |

High or low cfDNA tumor fraction is defined as patients whose cfDNA tumor fraction is higher or lower than the median of the cfDNA tumor fraction among all 20 samples pre-treatment. Platinum–sensitive was defined as disease progression ≥ 90 days after first-line platinum–based chemotherapy, and platinum-resistant as disease progression < 90 days or during first-line chemotherapy.

Abbreviations: PFS: progression free survival; CI: confidence interval; ref: reference.

**Table S3: Multivariate Cox regression analysis of overall survival (OS) between patients with high vs. low pre-treatment circulating free DNA fraction**

| **Factors** | **Hazard ratio** | **Standard error** | **P value** | **95% CI** |
| --- | --- | --- | --- | --- |
| Age at inclusion (years) | 1.01 | 0.04 | 0.71 | 0.94–1.09 |
| Sex  (ref: male = 1) | 0.78 | 0.45 | 0.25 | 0.25–2.43 |
| Platinum sensitivity  (ref: platinum sensitive = 1) | 1.95 | 1.26 | 0.30 | 0.55–6.94 |
| cfDNA tumor fraction  (ref: low = 1) | 26.3 | 31.4 | 0.010 | 2.52–273.9 |

High or low cfDNA tumor fraction is defined as patients whose cfDNA tumor fraction is higher or lower than the median of the cfDNA tumor fraction among all 20 samples pre-treatment. Platinum–sensitive was defined as disease progression ≥ 90 days after first-line platinum–based chemotherapy, and platinum-resistant as disease progression < 90 days or during first-line chemotherapy.

Abbreviations: OS: overall survival; CI: confidence interval; ref: reference.

**Table S4: Names of transcription factors (TFs) and the number of binding sites for TFs enriched in genomic regions with differential circulating cell-free DNA (cfDNA) occupancy post-treatment vs. pre-treatment**

| **Decreased cfDNA occupancy**  **post-treatment** | | **Increased cfDNA occupancy**  **post-treatment** | |
| --- | --- | --- | --- |
| **TF name** | **# sites** | **TF name** | **# sites** |
| EHF | 700 | Crx | 422 |
| ELF1 | 720 | Nkx2-5 | 665 |
| ELF4 | 603 | NRF1 | 1828 |
| ETV6 | 491 | REST | 1160 |
| KLF4 | 2382 | KLF4 | 3009 |
| MAFK | 1167 | PITX1 | 1692 |
| PLAG1 | 1493 | ZEB1 | 1452 |
| Six3 | 718 | TFAP2A | 2352 |
| SP2 | 1490 |  |  |
| TFAP2B | 1289 |  |  |
| TFAP2C | 2593 |  |  |
| VDR | 701 |  |  |
| Zfx | 2041 |  |  |
| ZNF263 | 1568 |  |  |

**References**

1. Adalsteinsson VA, Ha G, Freeman SS, Choudhury AD, Stover DG, Parsons HA, et al. Scalable whole-exome sequencing of cell-free DNA reveals high concordance with metastatic tumors. Nature communications. 2017;8(1):1324.

2. Cun Y, Yang TP, Achter V, Lang U, Peifer M. Copy-number analysis and inference of subclonal populations in cancer genomes using Sclust. Nature protocols. 2018;13(6):1488-501.

3. Favero F, Joshi T, Marquard AM, Birkbak NJ, Krzystanek M, Li Q, et al. Sequenza: allele-specific copy number and mutation profiles from tumor sequencing data. Annals of oncology : official journal of the European Society for Medical Oncology. 2015;26(1):64-70.

4. Gertz J, Savic D, Varley KE, Partridge EC, Safi A, Jain P, et al. Distinct properties of cell-type-specific and shared transcription factor binding sites. Molecular cell. 2013;52(1):25-36.
